# Clinical outcome analysis of frozen-thawed embryo transfer on Day 7

**DOI:** 10.3389/fendo.2022.1082597

**Published:** 2022-12-09

**Authors:** Xinmi Liu, Hua Lou, Junwei Zhang, Mingze Du, Yulin Du, Shanshan Wu, Yichun Guan, Jing Liu

**Affiliations:** Reproductive Medicine Center, The Third Affiliated Hospital of Zhengzhou University, Zhengzhou, China

**Keywords:** D7, FET, clinical pregnancy, live birth, abortion, euploid

## Abstract

**Objective:**

To investigate the clinical outcomes of Day 7 (D7) frozen-thawed embryo transfer (FET) and to provide a reference value for clinical work.

**Methods:**

This was a retrospective cohort study. Patients undergoing FET cycles in the Reproductive Medicine Center of the Third Affiliated Hospital of Zhengzhou University between December 2015 and January 2021 were included. According to the developmental stage of the embryos at transfer, the embryos were divided into three groups: Day (D) 5, D6 and D7 blastocysts. Group D7 was compared with Groups D5 and D6. Simultaneously, the preimplantation genetic testing (PGT) and non-PGT cycles in Group D7 were analyzed and compared. The main outcomes were the clinical pregnancy, live birth and miscarriage rates. The secondary outcomes were the implantation and euploidy rates.

**Results:**

In total, 5945, 4094 and 137 FET cycles were included in the D5, D6 and D7 groups, respectively. The clinical pregnancy rate was significantly lower in Group D7 than in Groups D5 (13.9% vs 62.9%, *P <*0.001) and D6 (13.9% vs 51.4%, *P <*0.001). Additionally, the live birth rate was significantly lower in Group D7 than in Groups D5 (7.3% vs 50.7%, *P <*0.001) and D6 (7.3% vs 40.5%, *P <*0.001). However, the miscarriage rate was significantly higher in Group D7 than in Groups D5 (47.4% vs 18.2%, *P* =0.001) and D6 (47.4% vs 20.6%, *P* =0.004). The clinical pregnancy and live birth rates for D7 blastocysts were significantly higher in the PGT group than in the non-PGT group (41.7% vs 13.9%, *P*=0.012; 33.3% vs 7.3%, *P =*0.003).

**Conclusions:**

D7 blastocyst transfer can yield a live birth rate that is lower than that for D5 and D6 blastocysts but has value for transfer. PGT for D7 blastocysts may reduce the number of ineffective transfers and improve the outcome of D7 blastocyst transfer, which can be performed according to a patient’s situation.

## Introduction

With continuous improvements in embryo culture systems, to improve the implantation rate of infertile couples (1), an increasing number of embryos are cultured to the blastocyst stage and then transferred. The number of days of blastocyst development represents the developmental potential of blastocysts (2) and affects the outcome of blastocyst transfer (3). The developmental potential of Day (D) 5, D6, and D7 blastocysts decreases gradually with the extension of culture time. Therefore, the conventional practice in the laboratory is to select blastocysts for transfer, biopsy or cryopreservation at D5 or D6 of embryo culture. This traditional standard was challenged in 2008 with the first report of successful pregnancy after vitrification cryopreservation of D7 blastocysts in humans (4).

At present, studies on D7 blastocysts are very limited and controversial. M.J. Gorrill et al. (5) argued that prolonging blastocyst culture to D7 did not increase the number of available embryos, and that the implantation rate of D7 blastocysts was very low, which would easily lead to adverse outcomes. Therefore, they did not advocate prolonging blastocyst culture to D7. However, recent studies (6, 7) have suggested that D7 blastocyst transfer has important clinical value, and approximately 3% of patients have usable blastocysts formed only on D7. Tiegs et al. (7) performed preimplantation genetic testing for aneuploidy (PGT-A) for 532 D7 blastocysts, screened out 229 D7 euploid blastocysts for frozen-thawed embryo transfer (FET), and found that the rate of continuous embryo implantation after D7 euploid blastocyst transfer is as high as 52.6%, which is similar to that for D5 or D6 euploid blastocyst transfer.

Clinically, among some patients, blastocysts fail to form at D5/D6 due to advanced age, poor ovarian function, poor embryo quality or other reasons, or blastocysts do form but only at D7, with fewer blastocysts formed at D5/D6. Therefore, the use of slow-developing D7 blastocysts will be of great significance for these patients. This article aimed to study the clinical outcomes of D7 blastocyst transfer and provide a factual basis for clinical practice.

## Materials and methods

### Study design

This retrospective cohort study was conducted at the Reproductive Medical Center of the Third Affiliated Hospital of Zhengzhou University. We enrolled all patients who underwent FET between December 2015 and January 2021.

Part I: The inclusion criteria were as follows: D5, D6, and D7 FET cycles. The exclusion criteria were as follows (1): a donor egg-assisted pregnancy cycle (2); repeated implantation failure (3); FET cycles on Day 2 (D2), Day 3 (D3) or Day 4 (D4) (4); FET cycles with sequential transfer (5); FET cycles with mixed embryo transfer with only one gestational sac implantation (6); preimplantation genetic testing (PGT) cycles; or (7) the absence of a delivery record or follow-up data (e.g., lost to follow-up). The embryos were divided into three groups according to the developmental day of the embryo at transfer: D5 blastocysts (D5 group), D6 blastocysts (D6 group) and D7 blastocysts (D7 group).

Part II: All D7 FET cycles were included, and D7 blastocysts were divided into preimplantation genetic testing (PGT) cycles and non-PGT cycles according to whether they originated from PGT cycles.

This study was performed in accordance with the basic principles of the Declaration of Helsinki. This study was approved by the Institutional Review Board of the Third Affiliated Hospital of Zhengzhou University.

### Controlled ovarian hyperstimulation

The conventional gonadotrophin-releasing hormone agonist (GnRH-a) (Diphereline, lpsen, France) long protocols of our center were used for ovulation stimulation (8), and eggs were collected 36-38 h after human chorionic gonadotropin (hCG) injection.

### Endometrial preparation protocols

The routine scheme of our center (9) was adopted for the endometrial preparation scheme of the FET cycle, which was selected according to the specific situation of the patient. At present, the commonly used schemes are mainly the natural cycle, artificial cycle and stimulation cycle. For patients with regular menstruation and normal ovulation, natural cycles were adopted. Artificial cycles were used for patients with anovulation, luteal insufficiency, and thin endometrium. Stimulation cycles were used in patients with follicular dysplasia, ovulation disorders, polycystic ovarian syndrome (PCOS), and contraindications to estrogen use.

### Insemination, embryo culture and embryo observation

IVF/ICSI insemination was performed 38-40 h after hCG injection, depending on the maturity of the egg and the processing time of the semen sample. An embryologist observed the fertilization of oocytes at 16-20 h after insemination. Sequential culture media were used for embryo culture (Vitrolife, Sweden; COOK, USA). After insemination, embryos were cultured in cleavage culture medium. On D3 after oocyte retrieval, the embryos were observed and scored. According to the patient’s own condition, 1-2 embryos with a cleavage stage grade of III or above were selected for fresh cycle transfer or cryopreserved. The remaining embryos were cultured with blastocysts, and then fresh-cycle blastocysts were transferred or cryopreserved according to the situation. The embryo was switched to blastocyst culture medium on D3 of culture, and the culture medium was not be changed again. On D5, D6, and D7, the development of blastocysts was observed and scored according to Gardner blastocyst classification standards (10). The blastocysts at stage 3 or above with an inner cell mass score ≥B were rated as blastocysts available for clinical transfer or freezing. Blastocysts with scores of 4BB and above were defined as high-quality blastocysts.

### Vitrified-warmed blastocysts

In order to avoid blastocyst hatching, start from D5 of the embryo, the development of blastocyst observation not only performance in the morning, but also in the afternoon. When stage 4 blastocysts are available, the laser is used to collapse and about 10 minutes later they are frozen. If the workload is heavy, the number of freezing personnel will be increased to ensure that the freezing process is carried out in strict accordance with the operation Standard Operation Procedure (SOP).

Vitrified-warmed reagents and carriers (Kitazato, Japan) were used. The reagents were removed from the refrigerator and equilibrated at room temperature for at least 30 min. The blastocysts were transferred to equilibrium solution (ES) for 10 min at room temperature and then transferred to vitrification solution (VS) for equilibrium. The blastocysts were placed on the labeled refrigerating carrier within 60 s. Then, the blastocysts were rapidly injected with liquid nitrogen and loaded into the cannula. At 37°C, the carrier cannula was removed, and the blastocyst ends were immediately immersed in thawing solution (TS) at 37°C for 1 min. Then, the blastocyst ends were transferred into diluent solution (DS) and then washing solutions 1 and 2 (WS1 and WS2) for 3 min. Finally, the cells were transferred into blastocyst culture medium (Vitrolife, Sweden) and placed in an incubator containing 6% CO_2_ at 37°C (tabletop incubator, Cook Company, USA) for transfer.

### Follow-up

Serum β-HCG levels were measured on the 14th day after transfer. For patients with positive serum β-HCG levels (≥50 IU/L), ultrasonographic assessment was performed on the 35th day after transfer. If the gestational sac was visible in the intrauterine cavity, clinical pregnancy was determined. The termination of pregnancy at less than 28 weeks of gestation with a fetal weight of less than 1000 g was considered a miscarriage. A live birth was considered if the pregnancy reached 28 weeks of gestation and a live neonate was delivered.

### Calculation of outcome measures

The outcome measures were calculated as follows: Clinical pregnancy rate = the number of clinical pregnancy cycles/the number of transfer cycles ×100%; Live birth rate = the number of live birth cycles/the number of transfer cycles ×100%; Abortion rate = the number of abortion cycles/the number of clinical pregnancy cycles ×100%; Implantation rate = the number of gestational sacs/the number of transferred embryos ×100%; and Euploidy rate = the number of euploid embryos/the number of embryos with PGT.

### Statistical analysis

All analyses were performed using Empower (R) (www.empowerstats.com, X&Y solutions, Inc. Boston MA) and R (http://www.R-project.org). Measurement data are expressed as the mean ± standard deviation (mean ± SD), and a t test (normal distribution) or Kruskal−Wallis rank-sum test (nonnormal distribution) was used for continuous variables. Categorical variables are represented as the number of cases (n) and percentage (%). The rate between groups was compared by chi-square analyses or Fisher’s exact test. *P*<0.05 was considered statistically significant.

## Results

### Comparison of basic clinical data and clinical outcomes among the three groups

A total of 10,176 FET cycles were included in this study, among which 5945, 4094 and 137 FET cycles were included in the D5, D6 and D7 groups, respectively. Compared with the D5 and D6 groups, in the D7 group, there were significant differences in infertility factors and endometrial preparation protocols (*P <*0.05). Compared with the D5 group, in the D7 group, there were significant differences in female age, male age, infertility duration, the average number of embryos transferred and the number of embryos transferred in the D7 group (*P <*0.05). The basal follicle-stimulating hormone (FSH) level in the D7 group was significantly different from that in the D6 group (*P*<0.05). The clinical pregnancy rate and live birth rate were significantly lower in the D7 group than in the D5 and D6 groups, and the differences were significant (*P*<0.001). The abortion rate was significantly higher in the D7 group than in the D5 and D6 groups (*P*<0.05) (**Table 1**).

**Table 1:** Comparison of basic clinical data and clinical outcomes among the three groups.

	Group D5 (n=5945)	Group D6(n=4094)	Group D7(n=137)	*P value*^a^	*P value*^b^
Female age (years)	32.4 ± 4.6	33.2 ± 5.0	33.4 ± 5.2	0.013	0.788
Male age (years)	33.3 ± 5.3	34.3 ± 5.9	34.4 ± 5.0	0.023	0.835
Female body mass index (kg/m^2^)	23.8 ± 3.3	23.8 ± 3.9	23.7 ± 3.1	0.878	0.875
Infertility type (%)				0.512	0.431
Primary infertility	2395 (40.3%)	1626 (39.7%)	59 (43.1%)		
Secondary infertility	3550 (59.7%)	2468 (60.3%)	78 (56.9%)		
Main infertility cause (%)				<0.001	<0.001
Female	3469 (58.4%)	2134 (52.1%)	46 (33.6%)		
Male	1039 (17.5%)	944 (23.1%)	67 (48.9%)		
Mixed	1109 (18.7%)	741 (18.1%)	24 (17.5%)		
Other	328 (5.5%)	275 (6.7%)	0 (0.0%)		
Infertility duration (years)	3.2 ± 2.6	3.4 ± 3.0	3.7 ± 3.1	0.023	0.318
Basal FSH (IU/L)	6.2 ± 2.3	5.2 ± 3.1	6.1 ± 2.2	0.520	<0.001
Endometrial preparation method				0.004	<0.001
Artificial cycle	2742 (46.1%)	2671 (65.2%)	45 (32.8%)		
Natural cycle	2190 (36.8%)	944 (23.1%)	68 (49.6%)		
Stimulated cycle	1013 (17.0%)	479 (11.7%)	24 (17.5%)		
Average number of embryos transferred (per patient)	1.193±0.395	1.311±0.463	1.314±0.466	0.003	0.947
The number of embryos transferred (per patient)				<0.001	0.947
1	4797 (80.7%)	2820 (68.9%)	94 (68.6%)		
2	1148 (19.3%)	1274 (31.1%)	43 (31.4%)		
Endometrial thickness (mm)	9.3 ± 1.6	9.4 ± 1.6	9.4 ± 1.9	0.556	0.575
Clinical pregnancy rate	3741 (62.9%)	2104 (51.4%)	19 (13.9%)	<0.001	<0.001
Miscarriage rate	681 (18.2%)	434 (20.6%)	9 (47.4%)	0.001	0.004
Live birth rate	3014 (50.7%)	1658 (40.5%)	10 (7.3%)	<0.001	<0.001

^a^represents Group D7 compared with Group D5, ^b^represents Group D7 compared with Group D6; Group D5 represents the D5 FET cycle; Group D6 represents the D6 FET cycle; Group D7 represents the D7 FET cycle.

### Basic clinical data and outcomes of FET cycles with D7 blastocysts from different sources

**Figure 1:**
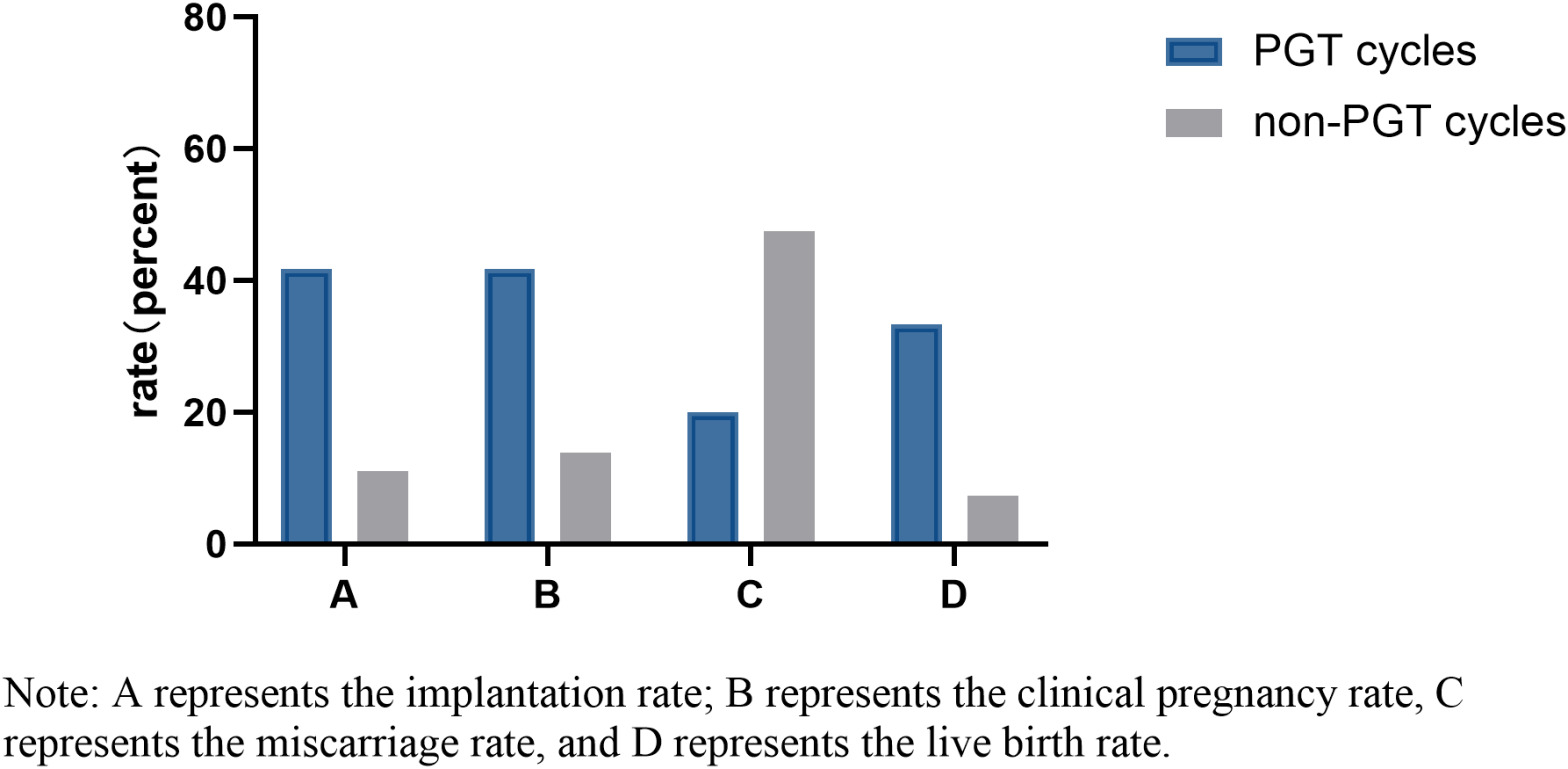
Clinical outcomes of D7 FET cycles from different sources.

Twelve and 137 D7 blastocysts in the PGT group and non-PGT group, respectively, were transferred. There were significant differences in infertility factors, basal FSH levels and the number of embryos transferred between the two groups (*P <*0.05). The implantation rate, clinical pregnancy rate and live birth rate in the PGT group were significantly higher than those in the non-PGT group, and the differences were significant (*P <*0.05). The abortion rate in the PGT group was lower than that in the non-PGT group, and the difference was not significant (*P*>0.05) (**Table 2**). The biopsy, report and transfer dates of twelve D7 euploid blastocysts are shown in **Table 3**. The clinical outcomes are also detailed in **Figure 1**.

**Table 2:** Basic clinical data and outcomes of FET cycles with D7 blastocysts from different sources.

	PGT cycles (n=12)	Non-PGT cycles(n=137)	*P value* ^c^
Female age (years)	34.2 ± 3.4	33.4 ± 5.2	0.561
Male age (years)	35.2 ± 3.1	34.4 ± 5.0	0.547
Female body mass index (kg/m^2^)	24.4 ± 2.2	23.7 ± 3.1	0.477
Infertility type (%)			0.513
Primary infertility	4 (33.3%)	59 (43.1%)	
Secondary infertility	8 (66.7%)	78 (56.9%)	
Main infertility cause (%)			<0.001
Female	3 (25.0%)	80 (58.4%)	
Male	6 (50.0%)	24 (17.5%)	
Mixed	0 (0.0%)	27 (19.7%)	
Other	3 (25.0%)	6 (4.4%)	
Infertility years (years)	4.2 ± 2.7	3.7 ± 3.1	0.548
Basal FSH (IU/L)	4.5 ± 1.6	6.1±2.2	0.013
Endometrial preparation method			0.228
Artificial cycle	6 (50.0%)	46 (33.6%)	
Natural cycle	6 (50.0%)	67 (48.9%)	
Stimulated cycle	0 (0.0%)	24 (17.5%)	
Number of embryos transferred (per patient)			0.021
1	12 (100.0%)	94 (68.6%)	
2	0 (0.0%)	43 (31.4%)	
Endometrial thickness (mm)	9.2 ± 1.4	9.4 ± 1.9	0.743
Implantation rate	5 (41.7%)	20 (11.1%)	0.009
Clinical pregnancy rate	5 (41.7%)	19 (13.9%)	0.012
Miscarriage rate	1 (20.0%)	9 (47.4%)	0.358
Live birth rate	4 (33.3%)	10 (7.3%)	0.003

c represents the comparison between PGT cycles and non-PGT cycles.

**Table 3:** Biopsy, report and transfer dates of 12 D7 euploid blastocysts.

ID	Biopsy date	Report date	Transfer date
I	November 20, 2018	December 20, 2018	January 18, 2019
II	November 12, 2018	December 10, 2018	April 7, 2019
III	April 02, 2019	May 1, 2019	May 30, 2019
IV	May 29, 2019	June 30, 2019	November 15, 2019
V	August 2, 2019	September 2, 2019	May 4, 2020
VI	October 12, 2019	November 10, 2019	July 31, 2020
VII	June 25, 2019	July 20, 2019	August 14, 2019
VIII	September 04, 2019	October 3, 2019	October 27, 2019
IX	May 19, 2019	June 18, 2019	November 12, 2019
X	September 04, 2019	October 1, 2019	December 7, 2019
XI	October 08, 2019	November 08, 2019	April 6, 2020
XII	May 20, 2020	June 18, 2019	July 20, 2020

### Comparison of the euploidy rates of D5, D6 and D7 blastocysts in the PGT cycles

The detection results of 2261 blastocysts from 581 cycles of PGT-assisted pregnancy in our center from January 2017 to December 2020 were included. The euploidy rate of D7 blastocysts was significantly lower than that of D5 blastocysts (*P*<0.001). The euploidy rate of D7 blastocysts was also lower than that of D6 blastocysts, but the difference was not significant (*P* =0.235) (**Table 4**).

**Table 4:** Comparison of the euploidy rates of D5, D6 and D7 blastocysts in the PGT cycles.

	D5	D6	D7	*P value*^d^	*P value*^e^
Total	896	1213	152		
euploidy rate				<0.001	0.235
No	459 (51.2%)	787 (64.9%)	106 (69.7%)		
Yes	437 (48.8%)	426 (35.1%)	46 (30.3%)		

^d^represents the comparison between D5 and D7 blastocysts, ^e^represents the comparison between D6 and D7 blastocysts.

### General conditions of live-born neonates and maternal complications during pregnancy after D7 transfer (including PGT cycles)

A total of 14 live births were obtained from D7 blastocysts, including 4 singleton live births in the PGT cycles and 9 singleton live births and 1 twin live birth in the non-PGT cycles. Ten neonates were male, and 4 neonates were female. The delivery mode was cesarean section in 8 cases and natural delivery in 6 cases. IDs 2, 4, and 9 were large for gestational age (LGA), 9 had macrosomia, and 10 was small for gestational age (SGA). In the PGT cycles, 1 premature newborn had neonatal respiratory distress syndrome (NRDS), and the mother had gestational diabetes mellitus (GDM). In the non-PGT cycles, one newborn was premature and had a good general condition; the puerpera had a twin pregnancy and experienced premature rupture of membranes (PROM). The overall prognosis of all neonates was good (**Table 5**).

**Table 5:** General conditions of live-born neonates and maternal complications during pregnancy after D7 transfer (including PGT cycles).

	ID	Number of live births	Gestational age	Newborn sex	Newborn weight (g)	Newborn length (cm)	Neonatal complications	Mode ofdelivery	Pregnancy complications
PGT cycles	1	1	28 w, 4 d	Female	1230	39	NRDS	Natural childbirth	GDM
	2	1	38 w, 2 d	Male	3800	50	Pathological jaundice	Cesarean	NA
	3	1	39 w, 3 d	Female	3300	50	NA	Cesarean	GDM
	4	1	39 w, 6 d	Male	3850	51	NA	Natural childbirth	NA
Non PGT cycles	5	2	35 w, 1 d	Male/ Male	2250/ 2250	48/48	Pathological jaundice/NA	Cesarean	PROM
	6	1	38 w, 1 d	Male	2900	50	NA	Natural childbirth	NA
	7	1	38 w, 5 d	Male	3200	50	NA	Natural childbirth	NA
	8		38 w, 5 d	Male	3460	51	NA	Cesarean	NA
	9	1	39 w, 4 d	Male	4100	52	NA	Cesarean	NA
	10	1	39 w, 5 d	Female	2600	49	NA	Cesarean	NA
	11	1	40 w	Female	3580	50	NA	Cesarean	Anemia during pregnancy
	12	1	40 w, 2 d	Male	3700	52	Pathological jaundice	Cesarean	GDM
	13	1	40 w, 6 d	Male	3400	50	NA	Natural childbirth	NA
	14	1	41 w, 4 d	Male	3360	54	NA	Natural childbirth	NA

NA represents not available.

## Discussion

In this study, a large amount of data and systematic observation and analysis were used to find that although the clinical pregnancy rate, live birth rate and euploidy rate of D7 blastocysts were lower than those of D5/D6 blastocysts and the abortion rate was higher, D7 blastocysts could still progress to healthy live births. At the same time, this study was the first to compare the clinical outcomes of D7 blastocysts derived from PGT cycles and non-PGT cycles and showed that the implantation rate, clinical pregnancy rate and live birth rate of PGT cycles were significantly higher than those of non-PGT cycles. This provides a factual basis for whether to use D7 blastocysts and how to make rational decisions in clinical practice.

The results of this study suggested that the parental ages of D7 blastocysts were significantly higher than those of D5 blastocysts, and the authors speculate that the advanced age of couples may be one of the reasons for the slow development of D7 blastocysts. Second, the infertility factors of D7 blastocysts were also significantly different from those of D5/D6 blastocysts. Among these factors, infertility in the D7 group was caused mainly by male factors, and male age may be one of the main factors. Related literature (11) suggests that increased male age is related to changes in epigenetic factors, and changes at the molecular level may affect the development of embryos. Due to the slow development and poor quality of D7 blastocysts, to improve the implantation rate of patients, mostly two blastocysts were transferred in non-PGT cycles. Therefore, the average number of embryos transferred and the number of embryos transferred as D7 blastocysts were significantly higher than those transferred as D5 blastocysts (**Table 1**).

The results of this study suggest that the transfer of D7 blastocysts can lead to a healthy live birth. When the average number of embryos transferred per cycle is 1.3, the clinical pregnancy rate is 13.9%, the live birth rate is 7.3%, and the abortion rate is 47.4%, which is consistent with the results recently published by Kevin S. Richter et al. (12). Kevin S. Richter et al. (12) studied the FET cycles of 59 D5, 268 D6 and 48 D7 blastocysts and found that the clinical pregnancy rate of D7 blastocysts was only 15%, which was significantly lower than that of D5/D6 blastocysts, and the clinical pregnancy rate was similar to that in this study. The study by Huang et al. (13) included more data, namely, 1961 D5, 4910 D6 and 413 D7 blastocysts. The results showed that the clinical pregnancy rate of D7 blastocysts was 32.9% and that the live birth rate was 26.6%, which were significantly lower than those of D5/D6 blastocysts, and the abortion rate was 19.1% higher than that of D5/D6 blastocysts. At the same time, D7 blastocysts were found to have an increased risk of very large for gestational age (VLGA). Their study mainly reported the obstetric outcomes of D7 blastocysts and did not describe the average number of embryos transferred per cycle in detail. Therefore, the authors speculate that the difference in the average number of embryos transferred may be responsible for the great differences in the clinical pregnancy rate, abortion rate and live birth rate between their study and ours. In addition, due to the small sample size (only 14 live births after D7 transfer), our study could not conduct specific statistical analysis on the outcomes of singleton pregnancies and listed only the specific situation of each live birth cycle.

Embryo aneuploidy is the main cause of implantation failure, spontaneous abortion, and embryo termination, among other adverse outcomes (14). The implantation rate, clinical pregnancy rate and live birth rate of D7 blastocysts are low, and one of the reasons for the high abortion rate may be the low euploidy rate and high aneuploidy rate of D7 blastocysts. In this study, the euploidy rate of D7 blastocysts was compared with that of D5 and D6 blastocysts during PGT cycles, and it was found that the euploidy rate of D7 blastocysts was only 30.3%. Su et al. (15) examined 151 D7 blastocysts and found that 55 D7 blastocysts were euploid, with a euploidy rate of 36.7%, which was significantly lower than that of D5/D6 blastocysts, consistent with our study results. Samer Alfarawati et al. (16) found that the rate of aneuploidy was higher among blastocysts with slow development, which was also consistent with our results. Another study (17) suggested that the slow development of D7 blastocysts might be caused by their aneuploid character.

Therefore, this study compared the outcomes of D7 blastocyst transfer from PGT cycles and non-PGT cycles and found that the implantation rate, clinical pregnancy rate and live birth rate of D7 blastocysts in PGT cycles were higher than those of D7 blastocysts in non-PGT cycles and that the abortion rate was significantly lower than that in non-PGT cycles. To date, no relevant literature has been published. Although PGT is used mainly by infertile women with chromosome abnormalities, monogenic diseases, repeated implantation failure, recurrent abortion and advanced maternal age (18, 19), embryos derived from PGT cycles have more “abnormal” possibilities than those from non-PGT cycles. In the PGT cycles, the center strictly followed the principle of single blastocyst transfer. However, in the non-PGT cycles, to improve the chances of pregnancy, the center still chose to transfer as many embryos as possible. Consequently, the number of embryos transferred in the non-PGT cycles was significantly higher than that in the PGT cycles. However, after genetic testing, the transfer of embryos diagnosed as “euploid” could still achieve a much higher live birth rate than the transfer of embryos derived from non-PGT cycles. This suggests that biopsy and genetic testing of D7 blastocysts can greatly improve the utilization efficiency of D7 blastocysts and reduce the number of ineffective transfers.

The question of whether preimplantation genetic testing for aneuploidy (PGT-A) should be performed routinely for D7 blastocysts is worth further consideration. PGT-A is used mainly for patients with advanced age, recurrent abortion, chromosomal abnormalities or other conditions. PGT-A is an invasive procedure that is used to improve the effectiveness of assisted reproductive technology (ART) and shorten the time to clinical pregnancy (19). Whitney et al. (20) believed that the ultimate goal of ART was to achieve a healthy live birth and supported routine PGT-A for D7 blastocysts. From this perspective, we need to consider not only the time, effort, and cost of embryo culture, biopsy, and genetic testing (15) but also a patient’s time, energy, and emotional and financial capacity. With the gradual introduction of ART into medical insurance in some areas of China, this perspective may become possible in the future, and more infertile couples will surely benefit from it.

In patients with indications for PGT, As long as the blastocyst is rated as usable, the biopsy operation will be carried out, and the genetic testing will be carried out one week later. After the test result is obtained, the patient will be notified by phone to determine the date of transplantation.

Our study has shown the value of PGT-A in improving the implantation rate, clinical pregnancy rate, and live birth rate after D7 blastocyst transfer. Therefore, D7 blastocyst culture may be considered for patients who must undergo PGT-A to improve their chances of a healthy live birth (15). PGT-A may be a useful tool for screening euploid embryos with slow development. Therefore, embryos that do not form usable blastocysts at D6 should not be generally discarded, and extending embryo culture may improve the clinical outcomes of some patients undergoing ART.

In order to improve the clinical pregnant rate of infertile patients, D7 blastocyst culture would be performanced for patients whose D5/D6 embryos did not form usable blastocysts. At this time, endometrial and embryo development were not synchronized, so we conducted FET for the D7 blastocyst. In this study, we further calculated that the live birth rate of D7 single blastocyst transfer was 7.1%, and the live birth rate of D7 double blastocyst transfer was 10.0%, indicating that the live birth rate of D7 double blastocyst transfer was higher than that of D7 single blastocyst transfer. Therefore, it is recommended to consider D7 double blastocyst transfer for patients without a PGT-A indication and with multiple D7 blastocysts. In contrast, for patients with no PGT-A indication and only single D7 blastocyst formation, the patients and their families should be informed of the low live birth rate, and the next treatment plan should be considered based on the individual situation of the patients.

In summary, although the D7 blastocyst clinical pregnancy rate and live birth rate are low and the abortion rate is high, specialists can consider extending the blastocyst culture time and attempting D7 FET when multiple cycles have failed to yield ideal embryos, D5/D6 embryos have not formed blastocysts (6, 15), the development of D6 embryos is unclear, or embryonic development is slow. PGT for D7 blastocysts may reduce the number of ineffective transfers and improve the outcomes of D7 blastocyst transfer, and this can be implemented according to the conditions of individual patients. D7 blastocyst culture is recommended for patients who must undergo PGT-A (16). It is suggested that double blastocyst transfer be prioritized for patients with D7 blastocysts without PGT-A indications. For patients with only one D7 blastocyst without PGT-A indications, it is recommended to carefully consider the next treatment plan. Additionally, one study (21) suggested the use of D7 blastocysts ≥180 μm in diameter to improve the clinical outcomes of D7 blastocyst transfer. Due to the limited data in our study, whether D7 blastocyst transfer has the risk of epigenetic, innate (22) or acquired developmental abnormalities is not known at present, and this needs to be examined by more rigorous prospective studies with a larger sample size.

## Data availability statement

The raw data supporting the conclusions of this article will be made available by the authors, without undue reservation.

## Ethics statement

This study was approved by the Institutional Review Board and approved by the Ethics Committee of The Third Affiliated Hospital of Zhengzhou University, which did not require informed consent. The ethics committee approved this study on August 19, 2022, and the approval number is 2022-219-01.

## Author contributions

XL, JL and HL contributed to the design of the study. XL, JL, JZ and MD performed the data extraction. YD and SW performed the statistical analyses. XL, JL and HL interpreted the data. XL, JL and HL wrote the manuscript. JL and YG contributed to the critical revision of the article. All authors contributed to the article and approved the submitted version.
